# PI16 is a shear stress and inflammation-regulated inhibitor of MMP2

**DOI:** 10.1038/srep39553

**Published:** 2016-12-20

**Authors:** Georgina G. J. Hazell, Alasdair M. G. Peachey, Jack E. Teasdale, Graciela B. Sala-Newby, Gianni D. Angelini, Andrew C. Newby, Stephen J. White

**Affiliations:** 1 School of Clinical Sciences, University of Bristol, Bristol Royal Infirmary, Bristol, BS2 8HW, UK; 2 School of Healthcare Science, Manchester Metropolitan University, John Dalton Building, Manchester M1 5GD, UK

## Abstract

Raised endothelial shear stress is protective against atherosclerosis but such protection may be lost at sites of inflammation. We found that four splice variants of the peptidase inhibitor 16 (PI16) mRNA are among the most highly shear stress regulated transcripts in human coronary artery endothelial cells (HCAECs), *in vitro* but that expression is reduced by inflammatory mediators TNFα and IL-1β. Immunohistochemistry demonstrated that PI16 is expressed in human coronary endothelium and in a subset of neointimal cells and medial smooth muscle cells. Adenovirus-mediated PI16 overexpression inhibits HCAEC migration and secreted matrix metalloproteinase (MMP) activity. Moreover, PI16 inhibits MMP2 in part by binding an exposed peptide loop above the active site. Our results imply that, at high endothelial shear stress, PI16 contributes to inhibition of protease activity; protection that can be reversed during inflammation.

Endothelial cells (ECs) are mechanosensitive, their behaviour being profoundly affected by both cyclic strain and the frictional force (shear stress) exerted by the blood flowing over their surface. ECs are exposed to a range of different shear stress environments *in vivo*, reviewed^1^,^2^,^3^. Disturbed/reversing flow, which produces low average and oscillatory shear stress (OSS), promotes an ‘activated and inflammatory’ EC phenotype that is proatherogenic: it also reduces antioxidant production, increases reactive oxygen species (ROS) production, encourages vascular leakage (by increasing the permeability of the endothelial cell barrier) and favours coagulation and thombosis. By contrast, normal laminar shear stress (LSS) (10–15 dynes/cm^2^ in the coronary circulation)^4^,^5^,^6^,^7^ induces EC quiescence, resistance to inflammation (by inducing a myriad of anti-inflammatory genes and repressing pro-inflammatory gene expression) and stimulates the release of anti-coagulative and anti-thombolytic mediators, all of which are atheroprotective^8^,^9^,^10^. In advanced atherosclerosis, ECs overlying stenotic atherosclerotic plaques are exposed to elevated shear stress (ESS), potentially exceeding 300 dynes/cm^2 ^5,7,11,12,13,14. However, the effects of ESS on ECs is less well studied. We previously reported that acute exposure to ESS (75 dynes for 24 h) modifies primary human umbilical vein EC behaviour compared to normal LSS, for example, altering MAP kinase signalling and reducing ROS levels and intracellular cAMP concentrations^15^. Similarly, Dolan and colleagues showed that exposing cultured bovine ECs to ESS of 10 Pa (100 dynes/cm^2^) for 24 h promotes the proliferation and pro-matrix remodelling behaviour, as well up-regulating the expression of anti-coagulant and anti-inflammatory genes^16^. Furthermore, the ECs overlying stenotic atherosclerotic plaques express markers of inflammation^17^,^18^, despite their exposure to ESS. The interaction of ESS and inflammation is therefore of particular pathological significance.

As we recently reviewed^19^, excessive extracellular proteolysis may also be a key factor underlying the loss of ECs during surface erosion from plaques, a phenomenon that can precipitate life-threatening myocardial infarctions. Extracellular proteases are also important regulators of EC attachment, migration and invasion, during angiogenesis^20^. Proteases are also essential in adaptive, EC-regulated arterial remodelling in response to flow^21^,^22^. For these reasons, our present study focussed on PI16 (also known as peptidase inhibitor 16, PSPBP, or CRISP-1), which is a member of the cysteine-rich secretory proteins, antigen 5, and pathogenesis-related 1 proteins (CAP) superfamily, and has homologues in other mammalian species including rat and mouse (reviewed in ref. 23). We found that PI16 is highly up-regulated by LSS and ESS in primary human coronary artery endothelial cells (HCAECs). However, PI16 is profoundly downregulated by pro-inflammatory cytokines. Using phage display we identified matrix metalloproteinase-2 (MMP-2) as a target for PI16 inhibition. Furthermore, we showed that PI16 inhibits EC migration.

## Results

### Laminar shear stress increased PI16 expression

**Figure 1:**
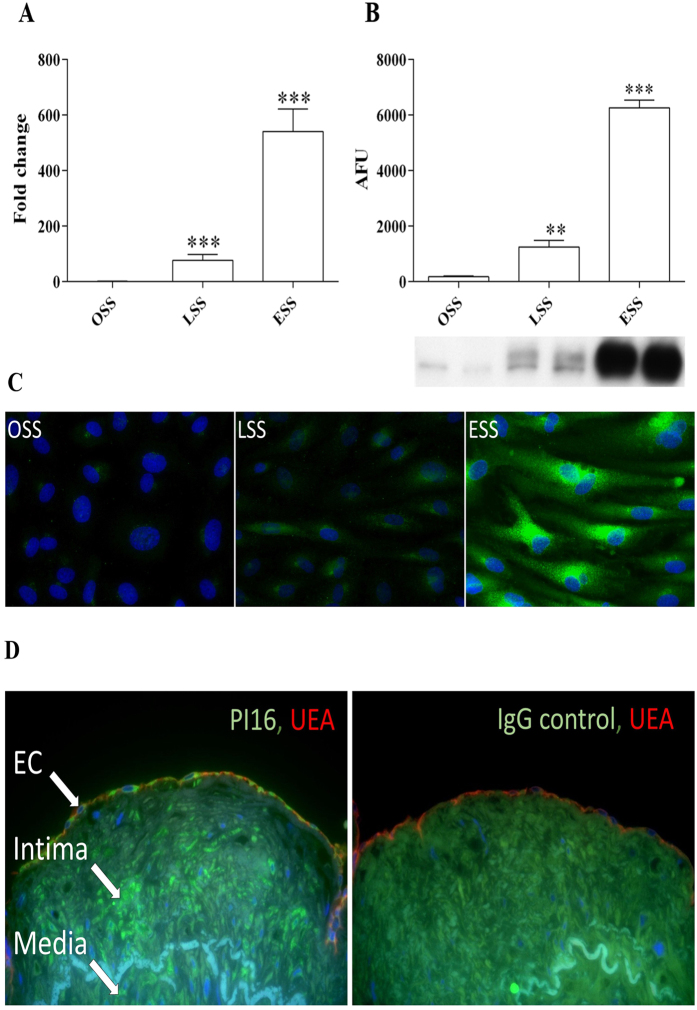
PI16 expression is increased by shear stress in HCAEC and is expressed in human coronary arteries. Reulation of mRNA (**A**) and protein (**B**) expression in HCAEC flowed for 72 h (n = 6; **P < 0.01, ***P < 0.001 compared to OSS control). (**C**) ICC staining corroborates western analysis, with highest PI16 expression (green) in cells exposed to ESS (blue DAPI stain highlights cell nuclei). (**D**) IHC analysis revealed PI16 staining (green) in ECs (endothelial cell marker, UEA in red). DAPI staining was used to demarcate the nuclei (blue).

Normal laminar shear stress (15 dynes/cm^2^ - LSS) and elevated laminar shear stress (75 dynes/cm^2^ – ESS) significantly increased the expression of PI16 transcript and protein in HCAECs compared with oscillatory shear stress (+/−5 dynes/cm^2^ – OSS) (Fig. 1A,B). LSS induced a 119-fold increase in mRNA (P < 0.001; Fig. 1A) and 7-fold increase in protein (P < 0.01; Fig. 1B). The expression was further elevated between LSS and ESS with a 9-fold increase in mRNA (P < 0.001; Fig. 1A) and a 5-fold increase in protein (P < 0.001; Fig. 1B). Results were confirmed with immunocytochemical staining of HCAEC exposed to shear stress, with a higher relative level of positive PI16 immunoreactivity in cells exposed to ESS when compared to LSS or OSS (Fig. 1C).

Four alternatively spliced PI16 mRNA variants have been previously identified, three that give rise to the full length 463aa PI16 protein (Supplementary Fig. 1, hereafter denoted as PI16 protein isoform 1) and a shorter 270aa isoform (isoform 2). Whilst cloning the PI16 gene for adenoviral construction, we identified a further two previously undescribed isoforms that are expressed by HCAECs, (hereafter referred to as PI16 protein isoforms 3 and 4) which translate into two distinct proteins (260aa and 225aa polypeptides respectively, see Supplementary Fig. 2). RT-qPCR analysis revealed that all of the isoforms were up-regulated in the same way by shear stress (Supplementary Fig. 3A).

### PI16 is expressed in HCAECs *in vivo*

Using histological sections of human coronary arteries from cadaveric donors, PI16 was clearly detected in luminal ECs (stained with UEA lectin) (Fig. 1D). Some of the cells in the intimal and medial smooth muscle cells were also positive for PI16 (Supplementary Fig. 4). IgG control sections were devoid of immunoreactivity.

### ERK5 signalling mediates upregulation of PI16 expression by elevated shear stress

**Figure 2:**
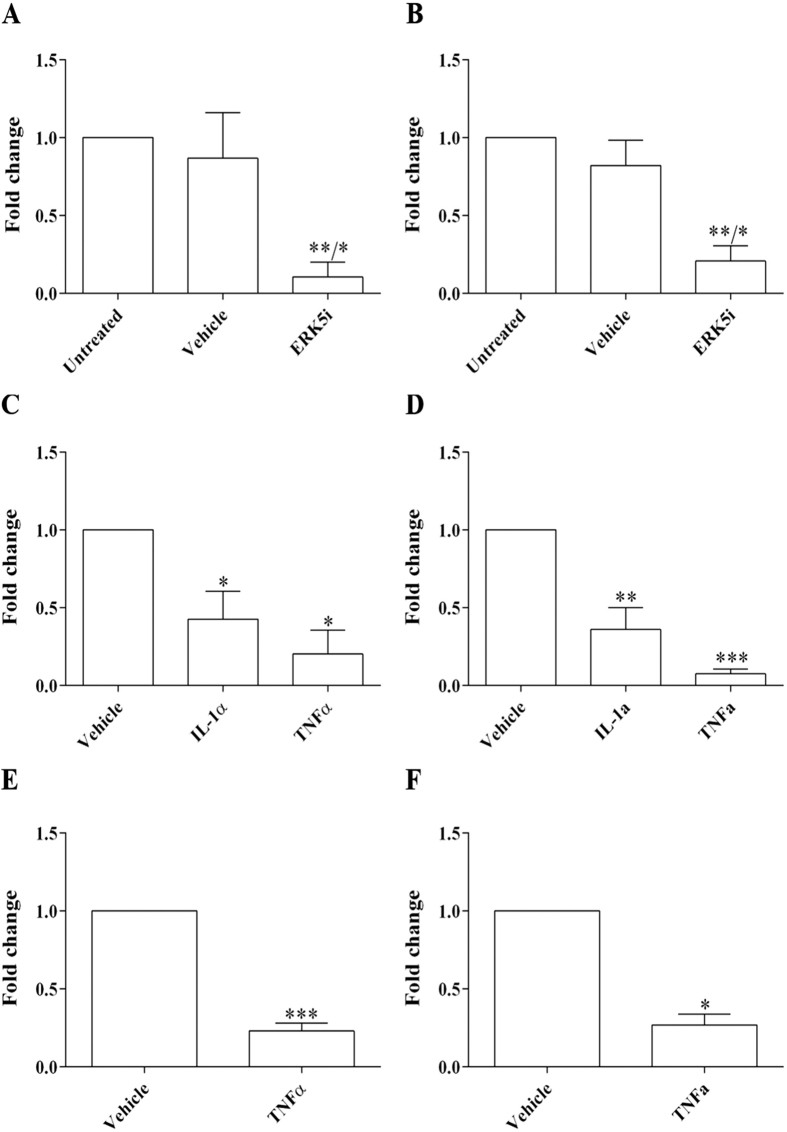
PI16 expression is regulated by ERK5 and modulated by inflammatory cytokines. To investigate whether ERK5 modulates PI16 expression, ESS was applied to HCAECs for 24 h in the presence of an ERK5 inhibitor BIX02189 (ERK5i; 10 μM). There was a significant reduction in PI16 mRNA (**A**) and protein (**B**) expression in HCAECs exposed to laminar shear when compared to the vehicle control (n = 3; *P < 0.05 compared to vehicle control, **P < 0.01 compared to untreated control). We also investigated the regulation of PI16 expression in the presence of inflammatory cytokines, TNFα and IL-1α, which significantly reduced PI16 mRNA (**C**) and protein (**D**) expression in HCAECs exposed to ESS for 24 h when compared with the vehicle controls (n = 3; *P < 0.05, **P < 0.01, ***P < 0.001). To exclude the possibility that cytokine treatment affected the adaptation of HCAECs to ESS, a further ‘chronic’ experiment was performed, HCAEC were exposed to ESS for 24 h prior to the first dose of TNFα. 3 doses of TNFα were added to the flow systems at 16 h intervals. TNFα treatment significantly reduced PI16 mRNA (**E**) and protein (**F**) expression (n = 3; *P < 0.05, ***P < 0.001) compared to vehicle controls.

Mitogen-activated protein kinase 7 (MAPK7), also known as Extracellular-Signal-Regulated Kinase 5 (ERK5), is strongly activated in response to shear stress. Moreover, the ERK5 signalling pathway has been shown to mediate some of the effects of LSS on ECs^24^,^25^,^26^. We therefore investigated whether ERK5 mediates PI16 upregulation in response to ESS. When HCAECs were exposed to ESS for 24 h in the presence of an ERK5 inhibitor, BIX02189 (ERK5i; 10 μM), the expression of PI16 mRNA (Fig. 2A; 8-fold reduction; P < 0.05) and protein expression (Fig. 2B; 4-fold reduction; P < 0.05) were significantly reduced in comparison to vehicle controls. This suggests that the shear-induced increase in PI16 expression is mediated by an ERK5-dependant pathway.

### Acute and chronic inflammation greatly reduces PI16 expression

We also investigated whether PI16 expression is regulated by atheroma-related pro-inflammatory cytokines, TNFα or IL-1α. Exposure of HCAECs to TNFα or IL-1α significantly reduced both the level of PI16 mRNA at ESS (Fig. 2C; TNFα, 5-fold reduction, P < 0.01; IL-1α, 2-fold reduction, P < 0.01) and protein expression (Fig. 2D; TNFα, 13.5-fold decrease, P < 0.05; IL-1α, 3-fold decrease, P < 0.05), when compared with vehicle controls. A similar reduction in PI16 mRNA and protein expression was observed at ESS for 72 h, with TNFα added for the last 48 h (to allow the cells 24 hours to adapt to their shear environment before treatment) (Fig. 2E,F).

### PI16 inhibits migration of HCAECs

**Figure 3:**
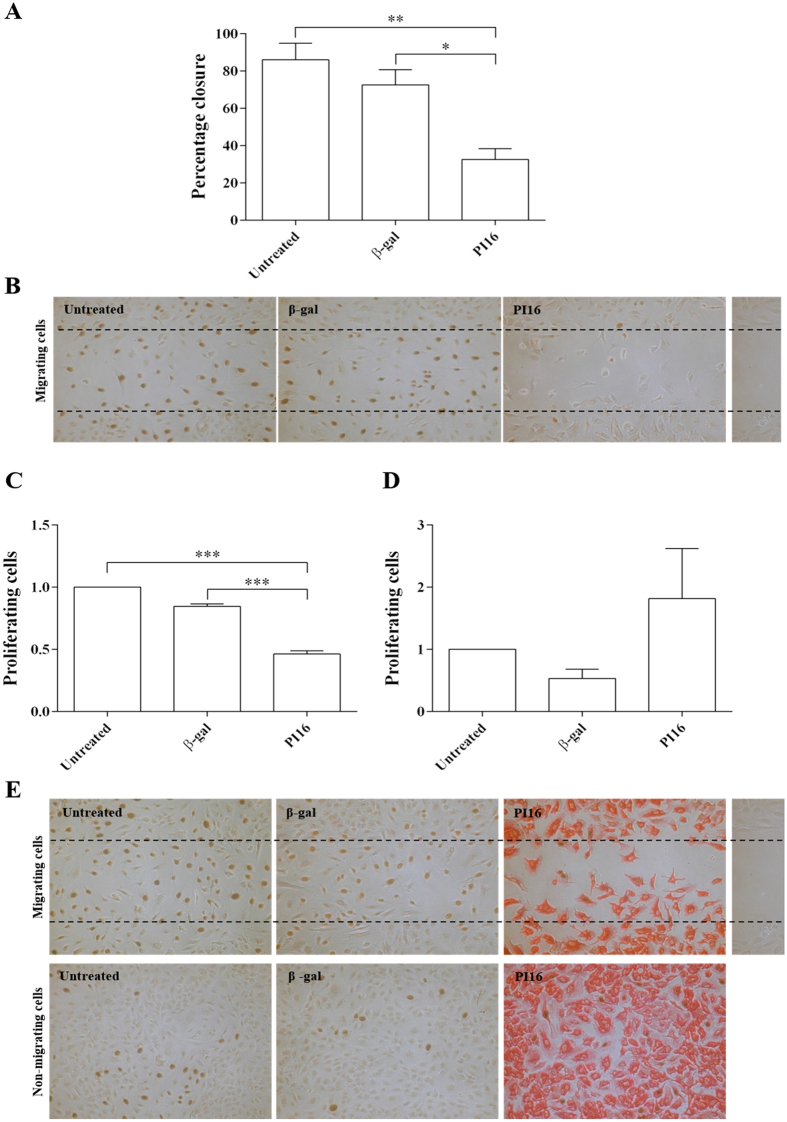
Effect of PI16 on migration and proliferation of HCAECs. Adenoviral-overexpression of PI16 significantly reduced the movement of HCAECs in a migration assay when compared to the β-gal adenoviral-controls (*P < 0.05 compared to β-gal control, **P < 0.01 compared to untreated control; n = 5) ((**A**) and representative image (**B**)). Staining for PI16 (red) revealed that the PI16-viral vector achieved ~100% cell transduction. Cells were also stained for BrdU to measure cell proliferation (brown). PI16 overexpression significantly reduced proliferation in the migration zone (**C**), ***P < 0.01 compared to controls), however did not affect the proliferation rate in non-migrating cells (**D**). (**E**) Representative images, BrdU staining in brown, PI16 staining in red.

Extracellular proteases are known to participate in cell motility^20^,^27^. Consequently, we hypothesised that adenoviral overexpression of PI16 would inhibit cell migration. Adenoviral overexpression of PI16 significantly reduced the migration of HCAECs into a ‘scratch wound’ *in vitro* when compared to β-gal transduced adenoviral control (40% reduction, P < 0.05; Fig. 3A). There was no difference in migration between β-gal adenoviral control and uninfected controls (Fig. 3A). Overexpression of β-gal or PI16 did not have any significant effect on the proliferation rates of HCAECs in confluent regions away from the scratch wound but PI16 overexpression significantly reduced proliferation in the migration zone (Fig. 3B). Cells were stained for PI16 to confirm transfection efficiency (Fig. 3E). PI16 immunoreactivity was detected in cultures infected with PI16 adenovirus but was absent in β-gal virus infected control cultures, as these experiments were performed under static culture conditions (Fig. 3E).

### PI16 overexpression reduces MMP activity in HCAECs

**Figure 4:**
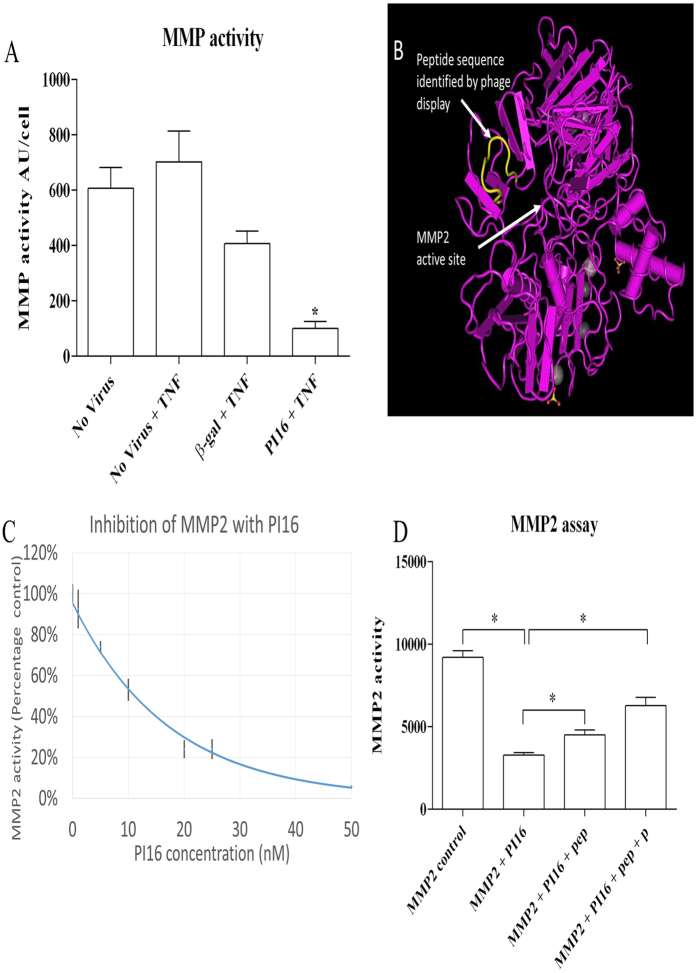
PI16 reduces MMP activity. (**A**) Concentrated conditioned media from control or PI16 over-expressing HCEACs was assessed for MMP activity using a quenched-fluorogenic substrate OmniMMP. In comparison to all controls, PI16 conditioned media showed the lowest MMP activity (*p < 0.05, n = 5). (**B**) Phage display identified a peptide containing the sequence TGPRSDGF, with high homology to amino acids 250–256 of MMP2 TG-RSDGF, corresponding to a peptide loop situated above the active site of MMP2. (**C**) Recombinant MMP2 (5.5 nM) was incubated with increasing concentrations of PI16 for 30 minutes before the addition of OmniMMP. Relative fluorescence 30 minutes after addition of OmniMMP is presented. Graph combines data from 3 separate preparations of recombinant PI16. Concentrations above 10 nM resulted in a significant reduction of MMP2 activity (P < 0.05, n = 3–23). (**D**) Inclusion of peptides TGRSDGF (pep) or TGPRSDGF (pep + P) (10 μM), with 10 nM PI16 reduced the inhibition of MMP2 (*p < 0.05 compared to all other conditions, n = 3).

It is known that matrix metalloproteinases (MMPs) are essential for endothelial migration^20^. Hence, we examined whether adenovirus driven PI16 production has the ability to inhibit MMP activity in cultures of HCAECs treated with TNFα to upregulate endogenous MMP release. Consistent with our hypothesis, there was a significant reduction of MMP activity in conditioned media from AdPI16 compared to Adβ-gal transduced HCAECs (Fig. 4A).

### Identification of MMP2 as a PI16 binding-partner using phage display

Phage display was used to identify peptides with high affinity for PI16, which might mediate binding and protease inhibition. Phage were isolated by cell surface bio-panning^28^,^29^ using HeLa cells (as they express low levels of PI16, by western blotting – not shown) infected with adenoviruses to overexpress PI16 or β-gal. Of particular interest, we isolated a peptide containing the sequence TGPRSDGF that had high homology with amino acids 250–256 (TG-RSDGF) of matrix metalloproteinase 2 (MMP2). These residues form an exposed loop adjacent to the active site, suggesting that PI16 may be an MMP2 inhibitor (Fig. 4B). Confirming this, recombinant PI16 produced in *E. coli* showed a strong inhibition of recombinant MMP2 activity (IC50 ~10 nM, Fig. 4C). Moreover, the phage-display-derived peptide TGPRSDGF or the corresponding sequence on MMP2, TG-RSDGF (lacks proline), reversed the inhibition of MMP2 activity by PI16, demonstrating that this peptide loop contributed to the binding of PI16 to MMP2.

## Discussion

Normal laminar shear stress initiates a phenotypic shift in ECs, promoting a more-quiescent, less-permeable, anti-inflammatory and athero-protective state. This study demonstrates that shear upregulates PI16 in HCAECs through activation of ERK5-dependent pathways. Moreover, PI16 inhibits endothelial migration and related proliferation, helping to maintain quiescence at high shear. PI16 up-regulation is reversed by inflammatory mediators, suggesting that this protective effect is lost in inflamed endothelium. Most importantly, MMP2 is identified for the first time as a target for PI16 inhibition.

We found that exposure to LSS for 72 h increased PI16 mRNA expression over 100-fold, compared to OSS. The magnitude of up-regulation was similar for all four splice variants that we identified in HCAECs, suggesting that shear regulates PI16 expression predominantly at the level of transcription. Protein expression mirrored the increases in mRNA level. Inhibition of PI16 mRNA and protein expression with the ERK5 inhibitor, BIX02189, suggests that ERK5 mediates shear upregulation of PI16, similarly to many other shear-regulated genes^24^,^25^,^26^. This observation would be strengthened by gene knock-down experiments. Furthermore, transcription factor Kruppel-like factor 2 (KLF2) is known to mediate many of the protective effects of shear that are downstream of ERK5^30^. Examination of supplementary gene array data from a study by Parmer *et al*.^26^ revealed that atheroprotective shear stress induced a 25-fold increase in PI16 expression in HUVECs compared to static controls, adding weight to our observations in HCAECs. This effect was reversed in the transcriptome after knock-down of KLF2, whereas the transcriptome observed after adenoviral overexpression of KLF2 showed a 64-fold increase in PI16 expression in HUVECs^26^. Although none of these findings were discussed by Parmer *et al*., they provide a convincing explanation for the shear regulation of PI16 expression.

Conversely, PI16 expression was strongly suppressed by inflammatory mediators, IL-1β and TNFα that act through the NF-κB signalling pathway. The mutual antagonism of pathways mediated by KLF2 and NF-κB has been extensively described previously^1^,^30^,^31^,^32^. Our studies extend this concept to expression of PI16.

It is possible to speculate on the relevance of these findings to human disease, particularly to endothelial erosion, a process responsible for ~30% of myocardial infarctions^33^. The upregulation of PI16 by KLF2 would suggest it plays an athero-protective role in the endothelium, by limiting proteolysis of the sub-endothelial matrix. This would help resist the increased mechanical forces to which the endothelium is exposed, especially overlying stenotic plaques, where the level of shear stress can exceed 300 dynes/cm^2 ^5,7,11,12,13,14. Cigarette use is a risk factor for endothelial erosion^33^ and is known to increase the circulating levels of inflammatory cytokines, including TNFα^34^. Downregulation of PI16 by circulating and plaque-derived inflammatory cytokines may therefore reduce protection from proteolysis and render the sub-endothelial matrix more susceptible to degradation and the endothelium to detachment.

Structural analysis of the full length PI16 protein suggests that, in addition to the hydrophobic cysteine-rich secretory (CRISP) domain, some isoforms contain a hydrophilic spacer domain and a putative single transmembrane domain (see Supplementary information and Supplementary Fig. 1). This implies that there are both cell surface expressed and secreted form of PI16.

PI16 was originally identified as a binding partner to the secreted protein microseminoprotein β (MSMB also known as PSP94)^35^. The function of PI16 in the prostate has not been identified but the serum levels of both PI16 and MSMB are negatively correlated with prostate cancer progression^36^, potentially implicating PI16 with inhibition of malignancy by MSMB^37^. PI16 is also strongly upregulated in human and rodent models of heart failure, where it accumulates in the intercellular space and inhibits murine cardiomyocyte growth^38^. Cardiac specific overexpression of full length PI16 reduced the size of cardiac myocytes, while inhibition of PI16 expression lead to myocyte hypertrophy, indicating a role for the regulation of cell size through a mechanism yet to be defined^38^. PI16 mRNA and/or protein has also been detected in a wide range of other tissues including the pituitary gland, tonsils, heart, small intestine, colon, prostate, testis and ovary^35^. However, until this current study, PI16 has not been shown to have any role in vascular biology. We found that PI16 inhibits migration and the related proliferation of ECs *in vitro*, suggesting that it promotes endothelial quiescence. We demonstrated that PI16 is expressed in the endothelium of human coronary arteries, consistent with our *in vitro* findings. PI16 was also present in other cells within the intima and media, suggesting other roles that may be the topic of future studies.

Until now, the function and physiological activity of PI16 has remained largely ill-defined. The demonstration that PI16 is a potent inhibitor of MMP2, with similar affinity to tissue inhibitors of metalloproteinases (TIMPs)^39^ is therefore of particular importance. MMP2 has long been known to participate in the invasion and migration of ECs during sprouting angiogenesis^20^. A similar function of MMP2 in migration and related proliferation of vascular smooth muscle cells during repair after vascular injury has also been demonstrated, along with a pathological role in models of aneurysm formation and atherosclerosis^40^. Inhibition of MMP2 by PI16 could therefore have a significant impact on several aspects of vascular pathology beyond endothelial dysfunction, as shown here.

MMP2 also plays a significant role in cancer^41^,^42^, MMP2 knockout mice show reduced tumour angiogenesis and tumour growth^43^ and expression of pro-MMP2 is increased in malignant cancers compared to benign cancers^44^,^45^, with the level of MMP2 expression providing utility as a prognostic marker^46^,^47^. In addition to extracellular actions, MMP2 has been shown to locate to pericellular^48^,^49^ and intracellular structures^50^, with its intracellular actions in cardiac myocytes having been shown to contribute to ischemia-reperfusion injury^51^,^52^,^53^. It is clear that MMP2 is an extremely significant protease and the identification of PI16 as a novel inhibitor of MMP2 highlights it as a potential regulator of many homeostatic and pathological processes.

## Materials and Methods

### Cell culture

Human coronary artery endothelial cells (HCAECs; >6 batches from different donors) were bought from Promocell (C-1222) and cultured in MV2 Medium (C-22121-Promocell). HCAECs were seeded onto gelatin-coated slides at high density and grown for 48–72 hours before initiation of flow. Cells were placed in a parallel plate flow apparatus as previously described^15^ and exposed to oscillatory flow (0 ± 5 dynes/cm^2^; OSS), normal laminar shear stress (15 dynes/cm^2^; LSS), or elevated shear stress (75 dynes cm^2^; ESS) for either 24 or 72 h. For 24 h shear stress experiments, ERK5 inhibitor BIX 02189 (S1531, SelleckBio 10 μM), TNFα (5ng/ml), or IL-1α (10 ng/ml) were added to media prior to the start of flow as previously described^15^. For 72 h shear stress experiments, cells were allowed to normalise to their respective shear stress environments for 24 h, before thee doses of TNFα (5 ng/ml) or control, were injected into the flow apparatus, at 16 h intervals (total treatment time of 48 h).

### Reverse transcription quantitative PCR (RT-qPCR)

RT-qPCR was performed using LightCycler^®^480 SYBR Green I master mix (Roche, UK) on cDNA prepared from HCAECs exposed to OSS, LSS, and ESS for 24 or 72 h. Standard curves were produced for each primer set and results calculated as copies of cDNA per ng total RNA. The primers used to detect all PI16 isoforms were designed based upon common sequences from the full length human PI16 gene (accession NM_153370.2; primer sequences in Supplementary Table 1).

### Western blotting

Following flow experiments, cells were lysed in SDS lysis buffer [2% SDS; 50 mM Tris pH 6.8; 10% glycerol]. Cell concentration in the lysate was quantified using PicoGreen (Invitrogen, Renfrew, UK) by mixing 100 μl of 1/1,000 cell lysate with 100 μl of 1/200 PicoGreen, both diluted in 10 mM Tris, 1 mM EDTA (pH 8). Fluorescence was measured with excitation at 485 nm and emission 520 nm and compared to a standard curve from a known cell number. 5000 cells were loaded per lane on denaturing SDS–polyacrylamide gels and blotted onto PVDF membranes. After blocking, membranes were probed with a primary rabbit anti-PI16 antibody (Sigma, UK-HPA043763; 1/3,000) overnight at 4 °C and detected with a HP-linked goat secondary antibody.

### PI16 immunocytochemistry and immunohistochemistry

HCAEC were exposed to shear stress before being fixed in cold 4% paraformaldehyde for 10 minutes. Cells were permeabilised with 0.1% triton, blocked in 20% goat serum and probed with rabbit anti-PI16 (1:100, Sigma - HPA043763), followed by goat anti rabbit Alexa Fluor 488 (1/200, Invitrogen). For immunohistochemistry, paraffin embedded human coronary artery sections were obtained from the Bristol Coronary Biobank (ethical approval 08/H107/48), dewaxed and rehydrated. Antigen retrieval was performed with citrate buffer (pH 6.0) incubated in a boiling water-bath for 30 minutes. Sections were blocked with 20% goat serum with the addition of pontamine sky blue (0.05%) to reduce background autofluorescence. Sections were incubated with rabbit anti-PI16 (1:100, Sigma - HPA043763) or rabbit IgG control, followed by goat anti rabbit Alexa Fluor 488 (1/200, Invitrogen). Rhodamine labelled Ulex Europaeus Agglutinin I (UEA, 1/200, Vector Laboratories RL-1062) was incubated with the primary antibodies to label endothelial cells.

### Cloning of PI16 and preparation adenoviral vector

PI16 was amplified with KOD polymerase (Merck Millipore) using primers SW653F and SW654R and cloned into a *NcoI*-deleted version of CpG-mcs (Invivogen) using the *BglII/NheI* restriction sites. The expression cassette from pCpG-mcs containing the mCMV enhancer, EF1α promoter, small synthetic intron, PI16 and polyA signal was removed by *EcoRI* digest and cloned into pDC511 (Microbix Biosystems, Canada). PI16 adenoviral vector (AdPI16) was produced using the Microbix Biosystems kit according to their protocols. Additionally, the *EcoRI* digest fragment containing the same expression cassette and β-galactosidase gene was removed from pCpG-LacZ (Invivogen) cloned into pDC511 and used as a vector-matched control (Adβgal). Viral stocks were amplified, CsCl banded, and titrated by plaque assay. AdPI16 gave an unusually low value on plaque assay when assessed at day 11 (normal day of calculation) however gave a much higher value when read at day 14 suggesting PI16 overexpression had an inhibitory effect on the plaque assay. We used immunocytochemistry to determine the relative pfu needed to achieve 100% transduction in HCAECs. Treatment with 25 PFU/cell of PI16 adenovirus lead to the infection of >95% cells Fig. 3E. Therefore, for subsequent experiments, HCAECs were infected with PI16 adenovirus with a multiplicity of infection of 25 plaque-forming units (PFU) per cell, we used a 4-fold higher pfu/cell for the Adβgal which gave an equivalent infection rate.

### Migration and bromo-deoxyuridine (BrdU) incorporation assays

The migration assay was performed using an Ibidi 2D invasion assay Culture-Insert (Ibidi) and proliferation was evaluated by BrdU incorporation. On day 1, cells were seeded at 1 × 10^4^ cells/partition into culture-inserts. On day 2, cells were transduced by either AdPI16 or Adβ-gal for 20 h. On day 3, media were changed. At 4 pm on Day 4, the culture-inserts were removed, leaving a 500 μm cell-free migration gap. Example pictures of the resulting 500 μm gap were taken using a light microscope (10x objective). 16 hours later on the morning of day 5, BrdU (10 μM, Sigma) was added to the media and the cells were left for a further 4 h. Cells were washed (ice-cold PBS) and fixed in 70% ethanol. The incorporated BrdU was detected by immunocytochemistry using a mouse anti-BrdU primary antibody (1/500, Sigma B8434), biotinylated goat anti-mouse secondary (1/250, Sigma) and Extravidin-HP (1/250, Sigma E2886), and visualised with diaminobenzidine staining. The cells were dual labelled with the rabbit anti-PI16 (1:150) antibody, signal developed with biotinylated goat anti-rabbit secondary (Sigma 1:250), and ExtrAvidin-Alkaline Phosphatase (Sigma, 1:250) and Fast Red TR/Naphthol AS-MX Phosphate (Sigma, UK). Multiple images of the wells were taken along the length of the cell-free gap for analysis of migration, as well as in the centre of each well for assessment of BrdU staining. For the migration assay, the distance between the two HCAEC populations were measured at 10 standardised points on each image. The percentage of cells positive for BrdU staining was calculated.

### Identification of PI16 binding peptides with phage display

Two populations of HeLa cells were generated by transduction with either Adβgal control or AdPI16 and used to isolate binding peptides, as described previously^28^,^29^. Briefly, 15 μl (~1 × 10^11^ pfu) of the phage library (random 12 amino acid library, New England Biolabs E8110S) was added to 1 ml 1% bovine serum albumin (BSA) in DMEM + HEPES (Invitrogen, UK), and incubated for 1 h at 4 °C on four successive cultures of Adβgal-transduced HeLa cells (HeLa-βgal). The cleared phage library was immediately incubated on HeLa cells infected with PI16-encoding adenovirus (HeLa-PI16), at 4 °C for 1 h. Cells were thoroughly washed (×5) with 1% BSA in PBS (with calcium and magnesium), weakly associated phage removed with 0.2 M glycine (pH 2.2), and cells lysed in 30 mM Tris, 1 mM EDTA (pH 8) to recover high affinity-bound phage. Subsequent biopanning rounds were performed with ~1 × 10^10^ phage, and included one pre-clearing step on HeLa-βgal cells prior to biopanning on HeLa-PI16 cells. After the completion of 4 rounds of biopanning, individual phage clones were amplified and screened for enhanced binding to PI16-transduced cells. Phage clones that demonstrated selective binding for PI16 were amplified, ssDNA prepared and sequenced. Peptide homology analysis was performed using BLAST.

### PI16 activity assay

Static HCAECs were infected with either β-gal- or PI16-expressing adenovirus. Following transduction with the virus, cells were washed twice with PBS and endothelial media (MV2 with growth factors but no serum) with TNFα (5 ng/ml) was incubated on the cells for 24 hours. Conditioned media were collected and concentrated (10x), cells were lysed in SDS lysis buffer and protein concentration assessed using the microBCA assay (Pierce). Protease activity of 5 μl concentrated conditioned media was assessed using OmniMMP (Enzo) fluorogenic substrate in MMP buffer [50 mM HEPES, 10 mM CaCl2, 0.15% Brij 35 pH 7.0], with 2 mM 4-aminophenylmercuric acetate (APMA). MMP activity was corrected for cell number by dividing the fluorescence by the protein concentration of the cell lysate.

Recombinant PI16 was generated by amplifying full length PI16 by PCR using primers SW773F and SW774R introducing an in-frame 5’BamHI site and 3′EcoRI site after the stop codon. PI16 was subcloned into pGEX-6P-1 bacterial GST fusion expression plasmid to facilitate the production of recombinant protein. Recombinant PI16 was expressed in SoluBL21 E. coli (AMS BIOTECHNOLOGY) in media comprising of 50% LB and 50% M9 media. Overnight culture was diluted 1:100 and grown at 37 degrees with shaking for 4 hours to an approximate OD of 0.8, after which it was cooled to 21 °C. PI16 expression was induced for 3 hs by the addition of IPTG at a final concentration of 1 mM. The bacterial pellet from 1 litre of culture was resuspended in 25 ml of PBS + protease inhibitor (Sigma P8340 at 1/100) before being pressure lysed in a Cell Disruptor (Constant Systems) at 25 kPsi. The lysate was cleared by centrifugation and concentrated using a 15 kDa filter centrifugation unit to a volume of 3 ml. The concentrated sample was then diluted back to 15 ml with PBS before being mixed with 500 μl of 4B GST binding sepharose (GE Healthcare) and incubated for 1.5 hs at 4 °C. The beads were then centrifuged at 500 g at 4 °C for 5 minutes and the supernatant was discarded. Beads where then washed 3 times with 1.5 ml of PBS. PI16 was eluted by thee sequential additions of 500 μl of 10 mM glutathione (G4251, Sigma). The eluted PI16 was dialysed (50 kDa MW cut off) into 50 mM HEPES, 10 mM CaCl2 and 100 mM NaCl. Protein concentration was measured using the microBCA assay (Pierce) and purity assessed using a SYPRO Ruby-stained polyacrylamide gel. In addition, competition for PI16 binding to recombinant MMP2 (Preprotech) was assessed using a direct activity assay with fluorogenic OmniMMP substrate (Enzo) using the phage display derived peptide TGPRSDGF or the MMP2 derived peptide TGRSDGF at 10 μM in MMP.

### Statistical analysis

Differences between groups were analysed using One-way Analysis of Variance (ANOVA), and either a Dunnett’s test to directly compare data with a single control group, or a Bonferroni or a Tukey-Kramer *post hoc* test for multiple comparisons. For two variables, groups were analysed using a two-way ANOVA and a Tukey-Kramer *post hoc* test. The type of analysis used for each experiment is indicated in the figure legends. In all instances, p < 0.05 was taken to be statistically significant.

## Additional Information

**How to cite this article**: Hazell, G. G. J. *et al*. PI16 is a shear stress and inflammation-regulated inhibitor of MMP2. *Sci. Rep.* **6**, 39553; doi: 10.1038/srep39553 (2016).

**Publisher's note:** Springer Nature remains neutral with regard to jurisdictional claims in published maps and institutional affiliations.

## Supplementary Material

Supplementary Data
