# Supplementary material for: PI16 is a shear stress and inflammation-regulated inhibitor of MMP2
Source: Sci Rep. 2016 Dec 20;6:39553. doi: 10.1038/srep39553 (PMC5171773; doi:10.1038/srep39553)
Supplement: Supplementary Data [file srep39553-s1.pdf]

PI16 is a shear stress and inflammation-regulated inhibitor of MMP2

Georgina G. J. Hazell, Alasdair M. G. Peachey, Jack E. Teasdale, Graciela B. Sala-Newby, Gianni D. Angelini, Andrew C. Newby and Stephen J. White

Supplementary information:

PI16 peptide sequence was analysed using several online prediction tools to determine its putative structure. Most of the tools used suggest the presence of a signal peptide (aa 1 to 28) and a terminal transmembrane domain (amino acids 442-463). The output from http://topcons.cbr.su.se/pred/ is below (Tsirigos KD, Peters C, Shu N, Käll L, & Elofsson A (2015) The TOPCONS web server for consensus prediction of membrane protein topology and signal peptides. Nucleic Acids Res. 43(W1):W401-W407.), showing the regions predicted to be inside the cell in red and outside in blue, then the consensus from the 6 different models underneath:

Predicted topologies and predicted ΔG values:

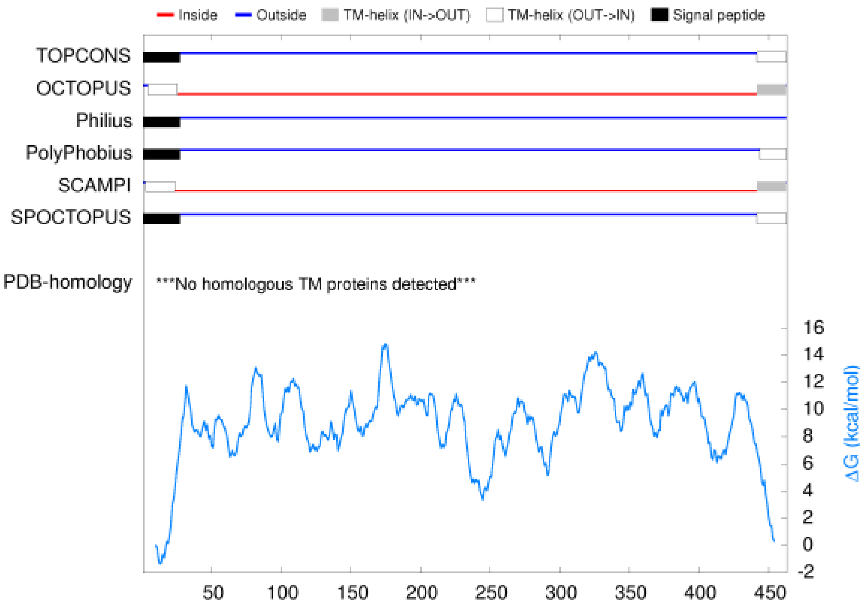

High-resolution image

Consensus prediction (TOPCONS):

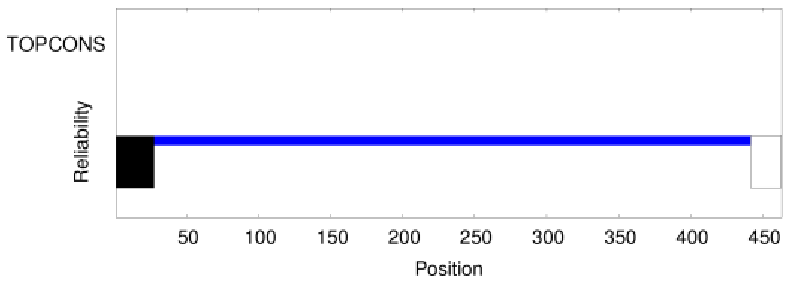

High-resolution image

Predicted signal peptide and TM-helix positions (position starting from 1):

TOPCONS	SP: 1-28,	TM1: 442-463
OCTOPUS	TM1: 5-26,	TM2: 442-463
Philius	SP: 1-28	
PolyPhobius	SP: 1-28,	TM1: 444-463
SCAMPI	TM1: 3-24,	TM2: 442-463
SPOCTOPUS	SP: 1-28,	TM1: 442-463
PDB-homology	***No homologous TM proteins detected***	

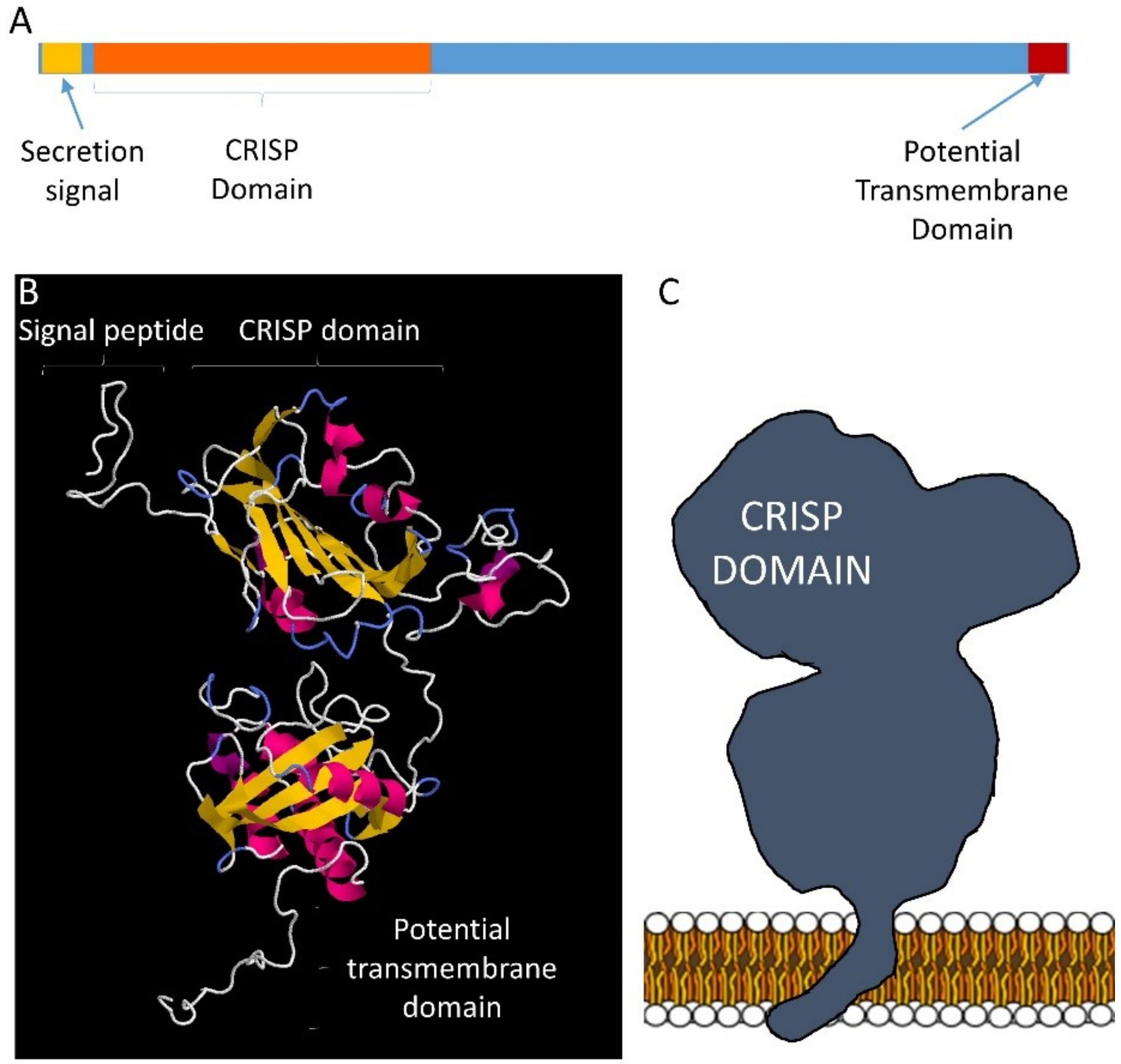

Supplementary Fig. 1. Putative tertiary structure of PI16. (A) Predicted PI16 domain structure. (B) Predicted protein structure of full length PI16, output from RaptorX prediction software http://raptorx.uchicago.edu/StructurePrediction/predict/ (Källberg M*, et al.* (2012) Template-based protein structure modeling using the RaptorX web server. *Nat. Protocols* 7(8):1511-1522.). (C) Based on a consensus from the prediction software, we have created a schematic of the putative structure of PI16.

Supp fig 2

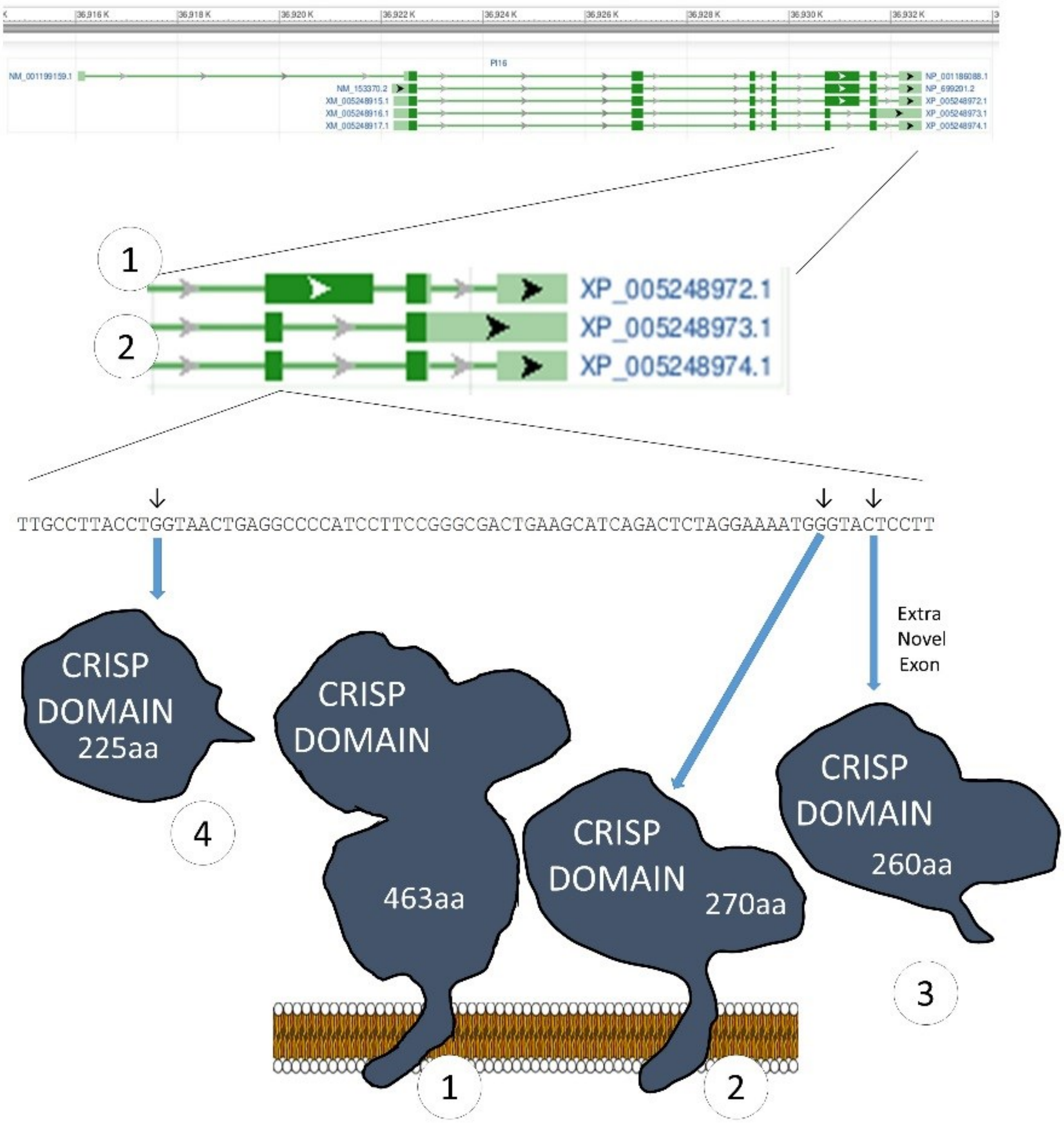

Supplementary Fig. 2. HCAECs express four PI16 isoforms. Four splice forms were isolated during the cloning of PI16. Isoform 1: the full length version. Isoform 2: a previously documented splice form that lacks the majority of exon 5, but uses a splice site that maintains the reading frame to retain the putative transmembrane domain. Isoform 3: is expressed at similar levels to isoform 1 and 2, but uses an alternative splice donor sites, including a further 4 base pairs that causes frame shift and alternative terminal amino acid sequence. Isoform 4: is the least abundant isoform in HCAECs (Supplementary Fig. 3, below), utilises an alternative proximal splice donor site and distal splice acceptor site with the same frame shift as isoform 3, thus sharing the same 12 terminal amino acids. With respect to isoform 3 and 4, an absence of the putative transmembrane domain, suggests these proteins may be secreted by endothelial cells.

Protein sequence of different splice forms of PI16. Sections of sequence are coloured to highlight regions lost during alternative splicing

```
>1 full length
MHGSCSFLMLLLPLLLLLVATTGPVGALTDEEKRLMVELHNLYRAQVSPTASDMLHMRWDEELAAFAKAYARQ
CVWGHNKERGRRGENLFAITDEGMDVPLAMEEWHHEREHYNLSAATCSPGQMCGHYTQVVWAKTERIGCGSHF
CEKLQGVEETNIELLVCNYEPPGNVKGKRPYQEGTPCSQCPSGYHCKNSLCEPIGSPEDAQDLPYLVTEAPSF
RATEASDSRKMGTPSSLATGIPAFLVTEVSGSLATKALPAVETQAPTSLATKDPPSMATEAPPCVTTEVPSIL
AAHSLPSLDEEPVTFPKSTHVPIPKSADKVTDKTKVPSRSPENSLDPKMSLTGARELLPHAQEEAEAEAELPP
SSEVLASVFPAQDKPGELQATLDHTGHTSSKSLPNFPNTSATANATGGRALALQSSLPGAEGPDKPSVVSGLN
SGPGHVWGPLLGLLLLPPLVLAGIF

>2 270aa
MHGSCSFLMLLLPLLLLLVATTGPVGALTDEEKRLMVELHNLYRAQVSPTASDMLHMRWDEELAAFAKAYARQ
CVWGHNKERGRRGENLFAITDEGMDVPLAMEEWHHEREHYNLSAATCSPGQMCGHYTQVVWAKTERIGCGSHF
CEKLQGVEETNIELLVCNYEPPGNVKGKRPYQEGTPCSQCPSGYHCKNSLCEPIGSPEDAQDLPYLVTEAPSF
RATEASDSRKMGAEGPDKPSVVSGLNSGPGHVWGPLLGLLLLPPLVLAGIF

>3 260aa
MHGSCSFLMLLLPLLLLLVATTGPVGALTDEEKRLMVELHNLYRAQVSPTASDMLHMRWDEELAAFAKAYARQ
CVWGHNKERGRRGENLFAITDEGMDVPLAMEEWHHEREHYNLSAATCSPGQMCGHYTQVVWAKTERIGCGSHF
CEKLQGVEETNIELLVCNYEPPGNVKGKRPYQEGTPCSQCPSGYHCKNSLCEPIGSPEDAQDLPYLVTEAPSF
RATEASDSRKMGTPSSLATGIPAFLVTEVQRALTSLASCQG

>4 225aa
MHGSCSFLMLLLPLLLLLVATTGPVGALTDEEKRLMVELHNLYRAQVSPTASDMLHMRWDEELAAFAKAYARQ
CVWGHNKERGRRGENLFAITDEGMDVPLAMEEWHHEREHYNLSAATCSPGQMCGHYTQVVWAKTERIGCGSHF
CEKLQGVEETNIELLVCNYEPPGNVKGKRPYQEGTPCSQCPSGYHCKNSLCEPIGSPEDAQDLPYLVQRALTS
LASCQG
```

```
3               MHGSCSFLMLLLPLLLLLVATTGPVGALTDEEKRLMVELHNLYRAQVSPTASDMLHMRWD
4               MHGSCSFLMLLLPLLLLLVATTGPVGALTDEEKRLMVELHNLYRAQVSPTASDMLHMRWD
1               MHGSCSFLMLLLPLLLLLVATTGPVGALTDEEKRLMVELHNLYRAQVSPTASDMLHMRWD
2               MHGSCSFLMLLLPLLLLLVATTGPVGALTDEEKRLMVELHNLYRAQVSPTASDMLHMRWD
                ************************************************************

3               EELAAFAKAYARQCVWGHNKERGRRGENLFAITDEGMDVPLAMEEWHHEREHYNLSAATC
4               EELAAFAKAYARQCVWGHNKERGRRGENLFAITDEGMDVPLAMEEWHHEREHYNLSAATC
1               EELAAFAKAYARQCVWGHNKERGRRGENLFAITDEGMDVPLAMEEWHHEREHYNLSAATC
2               EELAAFAKAYARQCVWGHNKERGRRGENLFAITDEGMDVPLAMEEWHHEREHYNLSAATC
                ************************************************************

3               SPGQMCGHYTQVVWAKTERIGCGSHFCEKLQGVEETNIELLVCNYEPPGNVKGKRPYQEG
4               SPGQMCGHYTQVVWAKTERIGCGSHFCEKLQGVEETNIELLVCNYEPPGNVKGKRPYQEG
1               SPGQMCGHYTQVVWAKTERIGCGSHFCEKLQGVEETNIELLVCNYEPPGNVKGKRPYQEG
2               SPGQMCGHYTQVVWAKTERIGCGSHFCEKLQGVEETNIELLVCNYEPPGNVKGKRPYQEG
                ************************************************************

3               TPCSQCPSGYHCKNSLCEPIGSPEDAQDLPYLVTEAPSFRATEASDSRKMG---------
4               TPCSQCPSGYHCKNSLCEPIGSPEDAQDLPYLV---------------------------
1               TPCSQCPSGYHCKNSLCEPIGSPEDAQDLPYLVTEAPSFRATEASDSRKMGTPSSLATGI
2               TPCSQCPSGYHCKNSLCEPIGSPEDAQDLPYLVTEAPSFRATEASDSRKMG---------
                *********************************

3               ------------------------------------------------------------
4               ------------------------------------------------------------
1               PAFLVTEVSGSLATKALPAVETQAPTSLATKDPPSMATEAPPCVTTEVPSILAAHSLPSL
2               ------------------------------------------------------------


3               ------------------------------------------------------------
4               ------------------------------------------------------------
1               DEEPVTFPKSTHVPIPKSADKVTDKTKVPSRSPENSLDPKMSLTGARELLPHAQEEAEAE
2               ------------------------------------------------------------
```

```
3       ------------------------------------------------------------
4       ------------------------------------------------------------
1       AELPPSSEVLASVFPAQDKPGELQATLDHTGHTSSKSLPNFPNTSATANATGGRALALQS
2       ------------------------------------------------------------


3       ------------TPSSLATGIPAFLVTEVQRALTSLASCQG--
4       -----------------------------QRALTSLASCQG--
1       SLPGAEGPDKPSVVSGLNSGPGHVWGPLLGLLLLPPLVLAGIF
2       ----AEGPDKPSVVSGLNSGPGHVWGPLLGLLLLPPLVLAGIF
                                        * .     *
```

The highlighted T represents a frequently observed polymorphism T50P caused by an A-C single nucleotide polymorphism of varying frequency and unknown significance, the 1000 genome frequency data is below:

ALL
A: 43%C: 57%

AFR
A: 22%C: 78% Sub-populations

AMR
A: 46%C: 54% Sub-populations

ASN
A: 46%C: 54% Sub-populations

EUR
A: 54%C: 46% Sub-populations

Supp fig 3

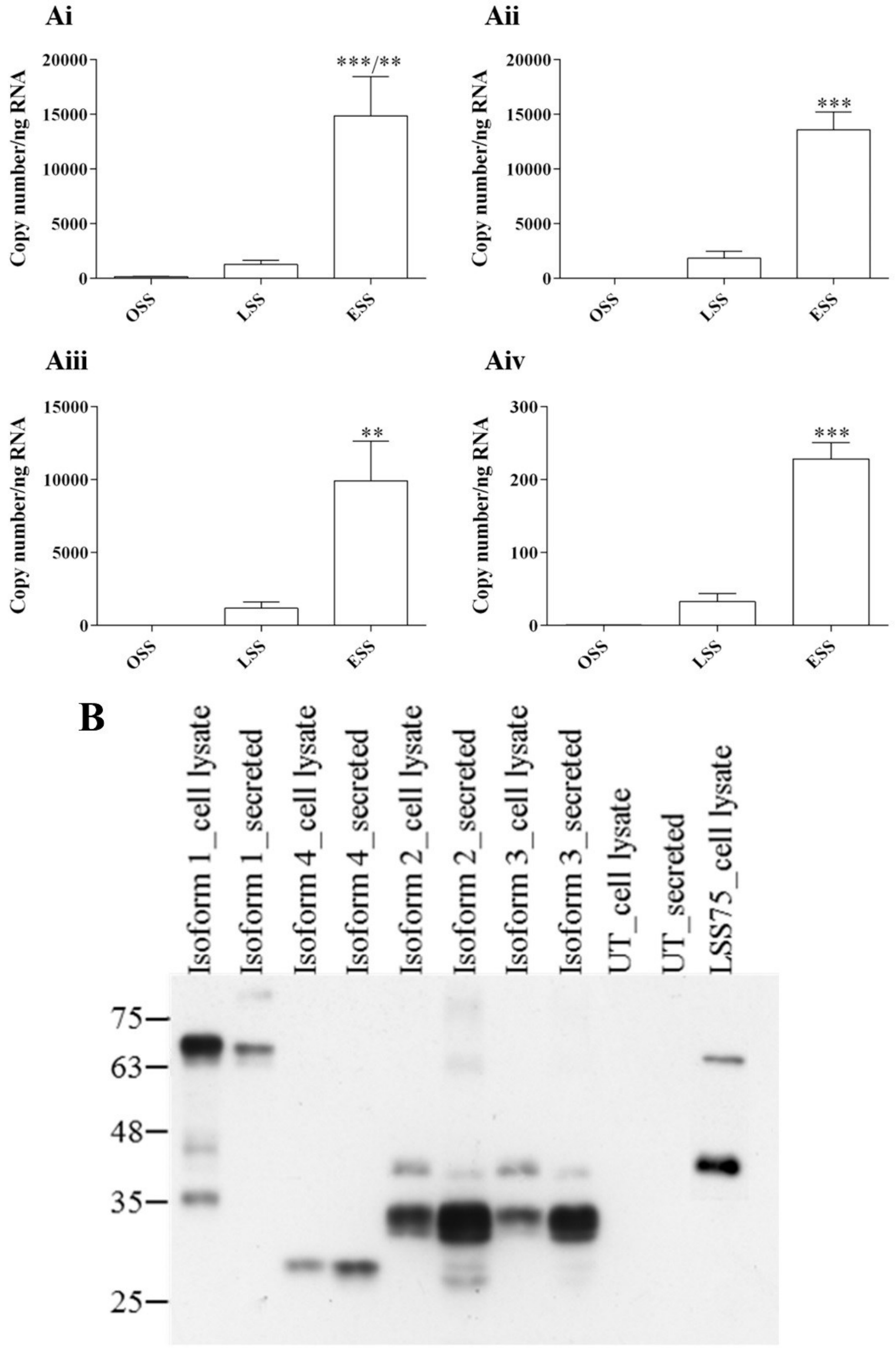

Supplementary Fig. 3. Analysis of isoform expression. (A) RT-qPCR analysis reveals that shear stress regulates the transcript expression of all four PI16 isoforms (Ai, isoform 1; Aii, isoform 2; Aiii, isoform 3; Aiv, isoform 4) expressed as copy number per ng of input RNA. (B) Western blot analysis cell lysates or conditioned media from CHO cells over expressing isoforms 1-4 by transient transfection, reveal the all four isoforms can be both cell bound and found in conditioned media. The blot also contains cell extract from HCAEC cells cultured under ESS (LSS75_cell lysate). Comparison of the CHO lysates with the HCAEC LSS75_cell lysate, suggests that the majority of the PI16 present in the HCAEC cell extract is the full length (molecular weight approximately 68KDa, isoform 1) but with an additional band at approximately 40KDa which may represent a larger molecular weight form seen in CHO transfections of isoforms 2 and/or 3. This is consistent with the qPCR data where isoform 1, 2 and 3 are highly expressed.

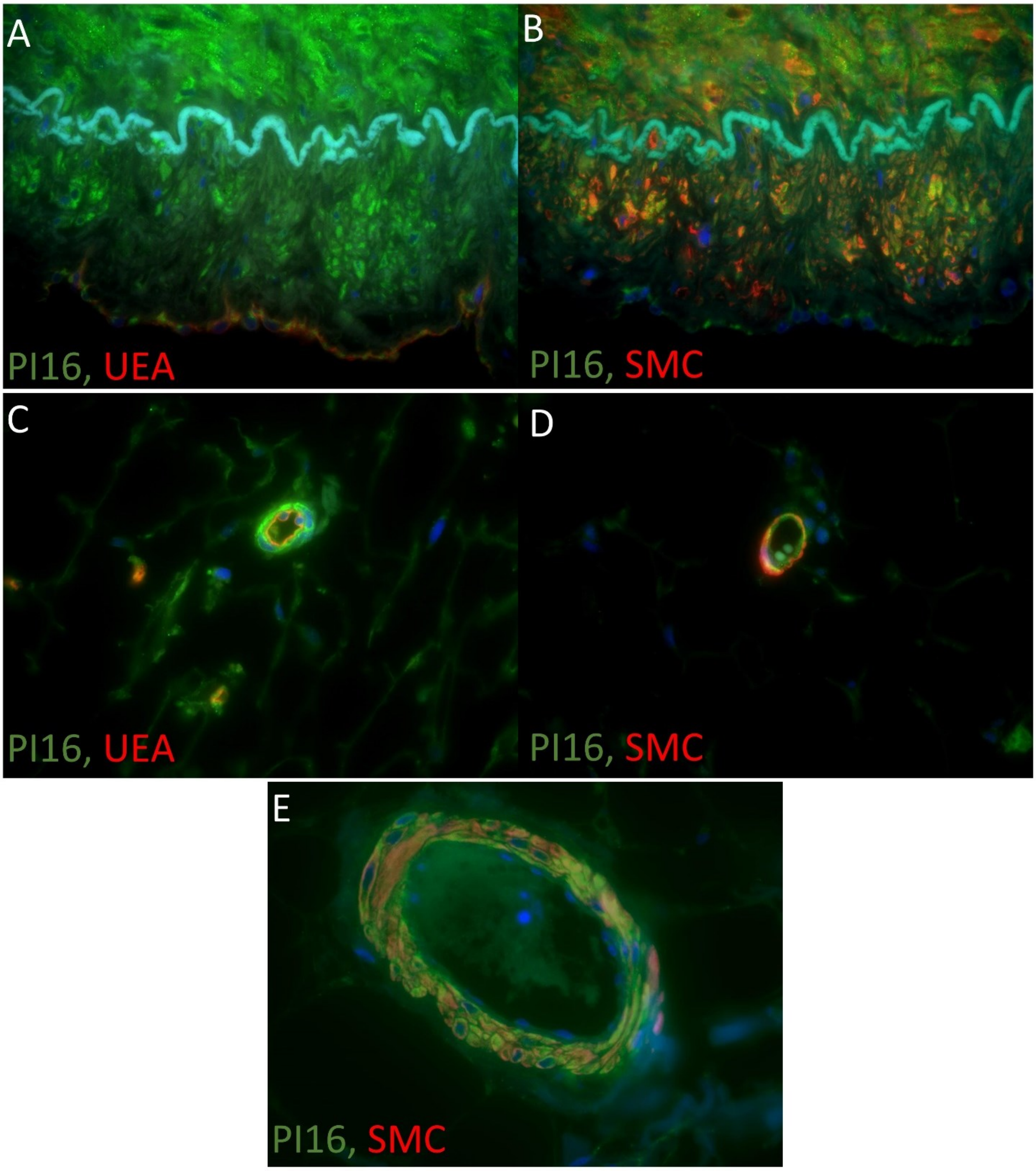

Supplementary Fig. 4. PI16 expression in human coronary arteries. Human coronary arteries were stained for PI16 and UEA as described in the methods. Additionally, serial sections were also stained with mouse anti-human smooth muscle cell actin (Dako, clone 1A4). The intimal cells that stained positive for PI16 were also SMC-actin positive; however not all intimal smooth muscle cells were positive for PI16 (B). C-E) Adventitial microvessel had comparatively low levels of PI16 staining in endothelial cells, with very strong staining in smooth muscle cells, suggesting microvascular endothelial cells do not express PI16 at the same level as large vessel endothelial cells.

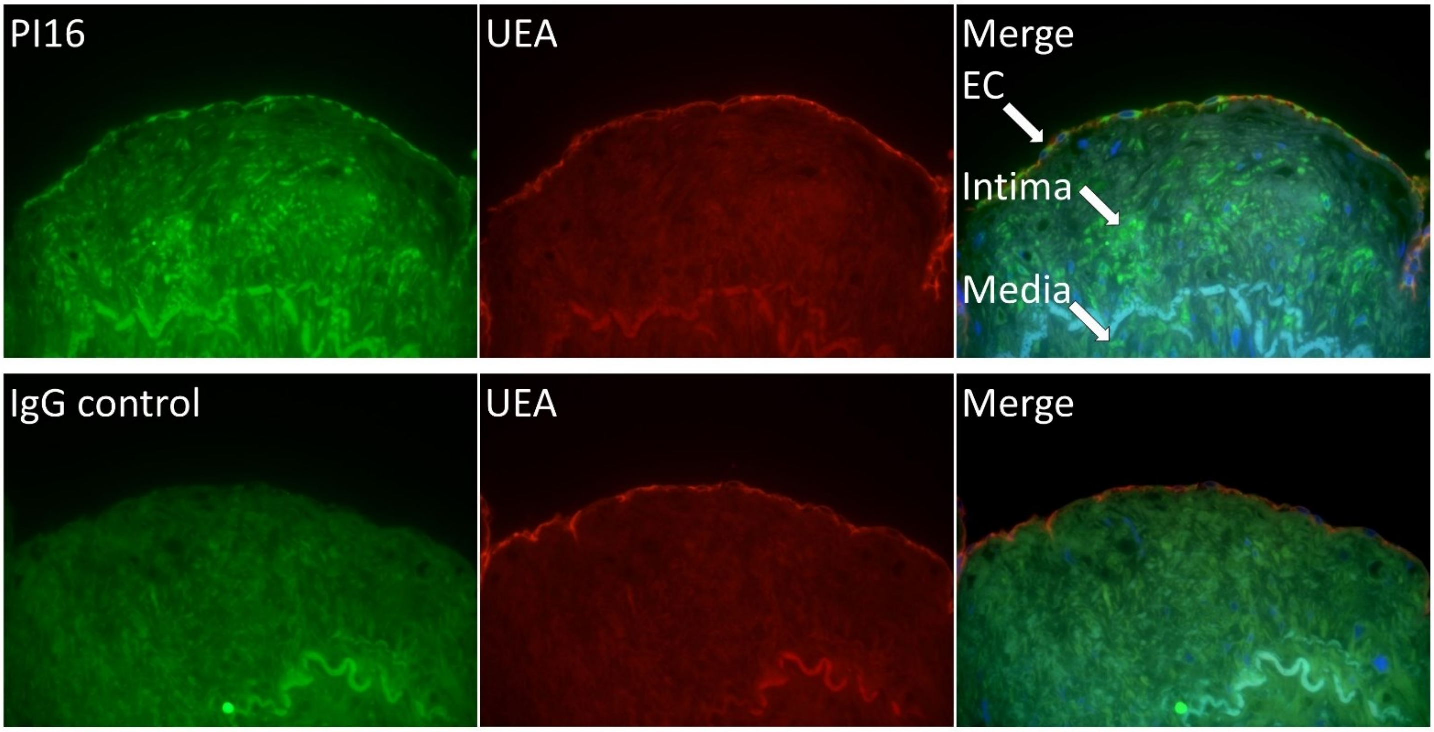

Supplementary Fig. 5. PI16 expression in human coronary arteries. Repeat of Figure 1C, showing individual channels and the merged image.

Supplementary Methods

Primer sequences:

PI16 primers

SW611F ATGTGCGGCCACTACACGCA

SW612R CCTTCACGTTCCCCGGAGGC

SW625F CCTCCGGGGAACGTGAAGGG

SW626R TCAGTCGCCCGGAAGGATGG

SW627F TAATGCCACGGGTGGGCGTG

SW628R TTCAGCCCTGACACGACGCT

Cloning primer: Forward creates BglII site and optimises Kozak sequence, reverse introduces NheI site:

SW653F GAGAGATCTCTGGCCACCATGCACGGCTCC

SW654R GAGTGCTAGCCCCTTCAGAAGATTCCAGCC

SW773F/SW774R PI16 cloning primers for N-terminal GST fusion introduces BamHI sites at both ends, 5' end in frame with coding sequence.

SW773F GATCTGGATCCTCCATGCACGGCTCCTGCAG

SW774R CCAGAATTCTAGCCCCTTCAGAAGATTCCAG

SW775F pGEX-6p-1 sequencing primer: GTATATAGCATGGCCTTTGC
